# The secret life of young adolescents living with HIV in northern Mozambique - a mixed methods study

**DOI:** 10.1186/s12889-021-11707-7

**Published:** 2021-09-10

**Authors:** Joana Falcão, Allison Zerbe, Claude Ann Mellins, Joanne Mantell, Kirsty Brittain, Bill Kapogiannis, Eduarda Pimentel de Gusmao, Teresa Beatriz Simione, Elaine J. Abrams

**Affiliations:** 1 ICAP at Columbia University, Mailman School of Public Health, New York, USA; 2 HIV Center for Clinical and Behavioral Studies, New York State Psychiatric Institute and Department of Psychiatry, Columbia University Irving Medical Center, New York, NY USA; 3 Division of Epidemiology & Biostatistics, School of Public Health & Family Medicine, University of Cape Town, Cape Town, South Africa; 4 Eunice Kennedy Shriver National Institute of Child Health and Human Development, Bethesda, MD USA; 5 National STI, HIV/AIDS Control Program, Maputo, Mozambique

## Abstract

**Background:**

In Mozambique, HIV infection remains a leading cause of adolescent mortality. With advances in antiretroviral treatment (ART), the population of adolescents living with vertically-acquired HIV is growing. Most studies of adolescents living with HIV (ALHIV) focus on older youth with horizontal infection. As part of a larger study, we examined the characteristics and health care needs of younger ALHIV, including those with vertically-acquired infection to inform preventive interventions.

**Methods:**

We used a convergent mixed-methods design and recruited ALHIV aged 12–14 years who were enrolled in HIV care in three health clinics in Nampula, Mozambique. From 11/2019–3/2020, we conducted 61 quantitative surveys and 14 in-depth interviews with a purposively selected subset of ALHIV who were aware of their HIV status. Descriptive statistical analysis was conducted for quantitative data. Qualitative data were transcribed and analyzed using thematic analysis.

**Results:**

The median age of ALHIV was 13 years, 50% were female, 67% lived with ≥1 parent, 70% had lost a parent, 100% were in school; 10% were in a relationship, and 3% had initiated sexual activity. Among 31 ALHIV aware of their serostatus, the median age of antiretroviral treatment (ART) initiation was 8 years (IQR: 6–11); 55% received caregiver support for ART management; 35% reported missing ≥1 ART dose in the last 30 days; 6% had disclosed their HIV-status to friends and 48% reported no one to talk to about HIV-specific issues. Four main themes emerged from the qualitative interviews with ALHIV: a) learning one’s HIV-positive status as the beginning of a secret life; b) importance of caregivers’ support for ART management; c) high value of ALHIV peer support to overcome isolation, increase HIV literacy, and support adherence; and d) unmet needs for sexual and reproductive health education.

**Conclusion:**

HIV-related secrecy prevails among ALHIV, a situation exacerbated by caregivers and healthcare providers. Caregivers play a major role in supporting adherence among young ALHIV, yet ALHIV could also benefit from adolescent-friendly services, including peer support, sexual and reproductive health services and preparation for independent health management. Integrating such programs into ART services in Mozambique may be critical to promoting ALHIV health.

## Introduction

Adolescents living with HIV (ALHIV) are a heterogeneous population and include individuals with both vertically- and horizontally-acquired HIV infection. In Eastern and Southern Africa there are currently 1.2 million ALHIV (10–19 years). With advances in antiretroviral treatment (ART) leading to global reductions in new pediatric HIV infections, as well as increased lifespan of those living with HIV, the population of adolescents with vertically-acquired HIV is growing. Unfortunately, there also is a high incidence of new horizontally-acquired infections among adolescents [1]. Although globally there have been great strides in increasing access to potent ART resulting in high levels of viral suppression among people on treatment in many countries [2], adolescents have consistently lower levels of ART initiation, viral suppression, retention in care and adherence than adults [3], warranting a focus on the needs of ALHIV during this developmental stage.

Adolescence is a period of accelerated growth and development across the trajectory from childhood to adulthood. It is characterized by enormous physical, cognitive, social, and emotional changes which differ during early (10–14 years) and late (15–19) adolescence. ALHIV face a particularly challenging period when parental influence wanes, risky behavior is not uncommon and the need to be with and liked by peers predominates [4]. To date, most research on the lived experience of adolescents with HIV has focused primarily on older youth, particularly those acquiring HIV infection from sexual partners. Less is known about the needs and experiences of younger adolescents, particularly those with perinatally-acquired infection who are entering this challenging period.

An estimated 140,000 ALHIV (10–19 years), including close to 50,000 younger adolescents were living with HIV in Mozambique in 2018 [1]; those aged 12–17 years accounted for 14% of the total population living with HIV [5]. Mozambique continues to be one of the countries reporting the lowest viral suppression rates for both children and adults [2]. While overall mortality among adolescents is decreasing in Mozambique, similar to other southern African countries, HIV remains one of the leading causes of death for adolescents [6].

However, interventions that target ALHIV, particularly the younger group, continue to be sparse in published literature, and there is an urgent need for larger and more rigorous studies [7, 8]. Interventions in other settings that have proven to be successful, such as Operation Triple Zero in Kenya and the Zvandiri CATS program in Zimbabwe, provide examples that have the potential to be adapted to the Mozambican context [9].

The CombinADO study is part of the PATC3H network [10], an initiative to generate scientific innovation to yield effective public health interventions for adolescents and young adults affected by HIV in low and medium income countries (LMICs) [11]. As part of the formative work to develop the CombinADO intervention, aimed at improving health outcomes (viral suppression, retention, and ART adherence) among ALHIV in Nampula, Mozambique, we explored the lived experiences of young ALHIV (12–14 years) obtaining services in three health facilities in northern Mozambique to enhance our understanding of the health needs of this population and inform intervention development.

## Methods

### Study design and setting

We used an explanatory convergent mixed-methods study design, with a cross-sectional quantitative survey and qualitative in-depth interviews (IDIs) collected and analyzed during the same time frame. Data integration occurred at the interpretation and reporting level using a narrative approach [12]. All methods were carried out in accordance with relevant guidelines and regulations under the Declaration section.

The study was conducted in Nampula City within Nampula province in northern Mozambique between November 2019 and March 2020. Nampula city is the third largest city in Mozambique located at the center of Nampula province, the most populous province in the country with 20% of the country’s total population. In 2020, there were 19 health facilities in Nampula City offering ART to approximately 40,000 patients. Our study was conducted at the three largest health facilities [13]. The survey and the IDIs were conducted during the first phase of the CombinADO study (UG3HD096926) [14].

### Sampling and recruitment

A cross-sectional survey was conducted among a sample of 61 dyads of ALHIV, aged 12 to 14 years, and their caregivers. Purposive sampling was used to recruit all available dyads of eligible male and female ALHIV and their caregivers receiving HIV services from the target clinics during the enrollment period. Written informed consent was obtained from ALHIV with consent from their adult caregivers. Following completion of the survey, we selected a subset of 14 ALHIV to participate in IDIs. ALHIV were invited to participate in an IDI based on having caregiver consent, being aware of their serostatus as confirmed with the caregivers, willing to participate, and being at ease talking about their HIV experience. Although there is no consensus as to the minimum number of participants for qualitative interviews, theoretical saturation is often used as a justification for sample size. In general, a sample that is relatively homogenous, like our sample, requires fewer number of cases [15]. In this study, we believe that thematic data saturation was achieved even with a small sample size as no new findings emerged in interviews conducted toward the end of data collection compared with interviews conducted in the beginning.

### Data collection

The same interviewers who administered the questionnaire, also conducted the IDIs, to facilitate trusting relationships with the participants. Both the survey and the IDIs were collected by a trained team of interviewers who were experienced in quantitative and qualitative data interviewing. Interviewers and participants were matched on sex Data collection was conducted in Portuguese. Participants received $5 USD at completion of the survey and an additional $5 USD for completion of the IDI as reimbursement for transportation.

#### Survey

Four interviewers administered the surveys in Portuguese via electronic tablets in private spaces in health facilities. The survey used questions adapted for young adolescents, translated into Portuguese and back-translated into English. We used Bronfenbrenner’s social-ecological model to guide assessment domains. Individual-level factors measured in the survey included demographic characteristics of the adolescent, medical history, health beliefs and knowledge, and reported ART adherence. We assessed knowledge about HIV using “true”/“false”/“don’t know” responses to general statements about HIV transmission, viral load and ART adherence. The interpersonal-level factors addressed relationships, sexual behavior, HIV disclosure and social support. The clinic-level factors addressed use HIV and of sexual and reproductive health services.

#### In-depth interview

The IDI guide explored adolescents’ perceptions related to HIV disclosure and stigma, ART adherence, coping and support, sexual and reproductive health. IDIs were conducted in a private space at the health facility, without a caregiver being present. The interviews were audio-recorded and transcribed verbatim and imported into QSR NVivo Version 12, a qualitative data analysis software program.

### Ethical considerations

The study was approved by the Mozambique Ethics and Scientific Review Committee (CNBS) and the Columbia University Irving Medical Center Institutional Review Board (CUIMC IRB). This research was also covered by a Certificate of Confidentiality from the US National Institutes of Health.

### Data analysis

We conducted descriptive statistics using STATA version 14.1 for the analysis of the survey data [16]. Deductive and inductive thematic analysis was used to analyze participants’ narratives in the IDIs [17]. Emergent codes were categorized under specific themes. We included the pre-existing themes, as “taking ART in the future” and “disclosure process” and newly identified themes as “disclosure brings humiliation” and “fear to talk with parents”. Transcripts were read several times to assess the consistency of the themes and sub-themes and to identify new relationships within and between themes. To interpret the main findings we compared and integrated results from the qualitative analysis with the quantitative results. To protect the confidentiality of participants and facilitate data presentation, we used fictious names for all participants.

## Results

### Demographics characteristics of all ALHIV

Sixty-one ALHIV (*n* = 30, 49% males) participated in the survey. All participants were enrolled in school and 67% were living with their mother or father. Few (15%) possessed their own phone or had ever used the internet. Median age at ART initiation was 8 years (IQR: 6–11). Seventy percent classified their health status as very good or good, 16% as fair and 13% as poor. Demographic characteristics of the 14 IDI participants were similar to those of the larger group of 61 ALHIV survey participants, but all were aware of their HIV status (Table 1).

**Table 1:** Demographic characteristics of ALHIV (*n* = 61)

	n	%
**Males**	30	49
**Aware of his/her serostatus**	31	51
**Age**, median [IQR]	13 [12–14]	
**Age at HIV diagnosis**, median [IQR]	8 [4–11]	
**Age at ART initiation**, median [IQR]	8 [6–11]	
**Currently lives with**		
Mother/Father	41	67
Grandmother / grandfather	9	15
Siblings	41	67
Other family members	9	15
Other	26	43
**Religion**		
Christian	39	64
Muslim	20	33
Other	2	3
**Has own cell phone**	9	15
**Has accessed the internet**	9	15
**Current school enrollment**	61	100
**Self-reported health status:**		
Excellent/very good	5	8
Good	38	62
Fair	10	16
Poor	8	13

### Overview of themes

**Fig. 1:**
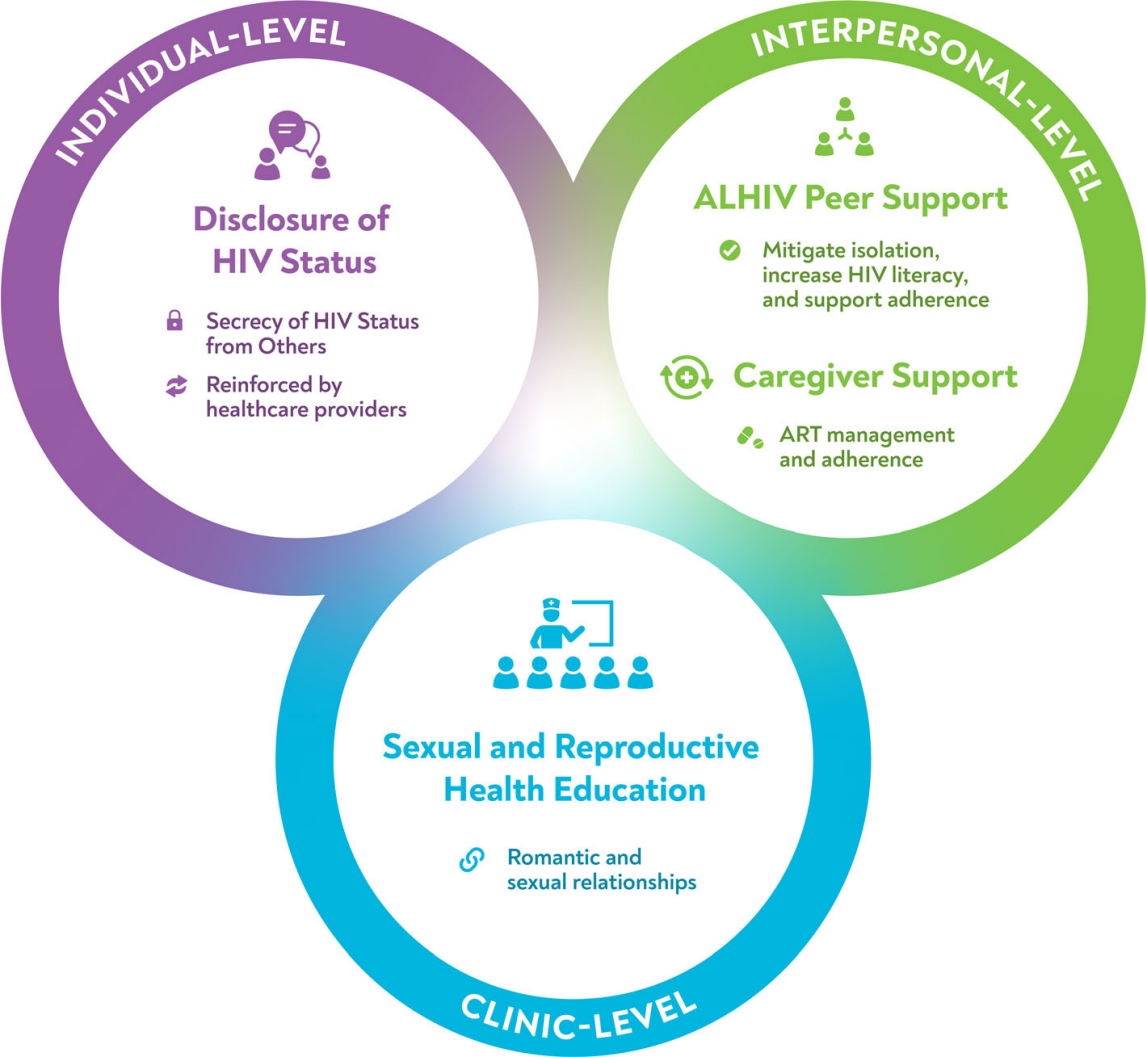
Diagram with Interrelationships across the four themes

Four main themes emerged from the qualitative data and we first summarize these findings followed by relevant survey data using *a narrative weaving approach* to integration and interpretation of the qualitative and quantitative findings [12]. We present also a diagram showing the interrelationships across the four themes and the three levels of Bronfenbrenner’s social-ecological model (Fig. 1).

### Theme 1: learning one’s HIV positive status marks the beginning of a secret life

A central theme that emerged from the IDIs was one of secrecy: the experience of learning their HIV-positive status marked the beginning of a secret life, because being HIV positive was a secret that must be kept from their friends and most of their family members. The disclosure moment took place either at the health facility or at home and was facilitated by either a caregiver, a healthcare provider or both. Regardless of the location and who was involved in this process, the majority of the ALHIV reported that along with being informed about their HIV status, they were also instructed about the importance of keeping this a secret from other people. This was reinforced both by the healthcare providers and the caregivers:

Interviewer: “Can you tell me how you knew you have HIV?

G: The nurse told me.

Interviewer: On the day the nurse spoke with you, you were with whom?

G: With my father.

Interviewer: And what did the nurse tell you?

G: She told me to take the pills [ARVs].

Interviewer: What else?

G: Not to mention to anyone that I have HIV.

Interviewer: What else?

G: Only that.

Interviewer: And how did you feel?

G: I felt sad.” (Gaspar, 13 years).

This secrecy was described as important to protect ALHIV from being humiliated and ostracized by their closest friends:

“Daddy tells me not to tell anyone, he told me not to mention that I have HIV because they (my friends) will stop playing with me.” (Joao, 13 years).

“The nurse told me not to tell anyone, not even the people I play with, otherwise they can they advantage of me. They can tell others and they won’t play with me.” (Isabel, 13 years).

Not surprisingly, none of the ALHIV interviewed had yet disclosed his/her serostatus to others and they were afraid of the consequences of doing this:

Before when I had a disease, I used to talk to my friends, now I can’t speak, I must hide it, nobody can know […] You cannot publish that you have this virus, that is for you to keep alone and nobody to know, because if someone else knows, they will tell other people and you will be humiliated.” (Rui, 14 years).

“I’m afraid that they may discriminate against me, treat me badly, or not want to play or talk to me, that’s why I want no one to know about my illness.” (Noemia, 13 years).

Although, when asked about future intentions to disclose, two-thirds of those interviewed stated that 1 day they intend to share their status with their romantic partners or close family members:

[In the future] “I intend to tell my husband because he must know, if I will not tell him, he will find out, he could follow me in the hospital and ask me why I am taking these pills, or he could search my purse and find the pills.” (Filipa, 13 years).

In addition, even though all of those interviewed were aware of their serostatus, only half of them understood how they acquired HIV infection:

Interviewer: “Did someone explain to you how you got infected with HIV infection?

A: No.

Interviewer: Can you tell me if you were born with HIV infection or got it afterwards?

A: I don’t know.

Interviewer: Didn’t they explain anything to you?

A: No.” (António 12 years).

The challenges around HIV infection disclosure and this secrecy were also evident in survey responses (Table 2). Questions around HIV status disclosure were only asked to 51% of the ALHIV that had been disclosed and hence were aware of the HIV-positive status. Among these, 39% had learned their HIV status at home and 58% at the clinic but the majority (87%) in the presence of a caregiver. All participants reported that their main caregiver was aware of their HIV status, but 45% reported that none of the other household members were aware. Almost all participants (94%) reported that none of their friends knew they were living with HIV. When asked about experiences of stigma and discrimination, 87% felt they were “a person of worth” despite their HIV status; however, 48% said they understood why people would reject their friendship because of their HIV infection, and 35% felt embarrassed about their HIV status.

**Table 2:** HIV status disclosure and stigma experiences among ALHIV (*n* = 31)

	n	%
**Adolescent knows his/her HIV status**	31	51.0^a^
**Remember when first learned HIV status** (*n* = 31)	21	68.0
**Age when learned HIV status**, median [IQR] (*n* = 21)	12 [11,12]	
**How was HIV status learned** (*n* = 21)		
Told by family at home	12	39.0
Told by doctor/nurse at clinic	3	10.0
Told by family and doctor/nurse at clinic	15	48.0
Don’t remember/other	1	3.0
**Other household members with HIV** (*n* = 31)		
Mother	9	29.0
Father	4	13.0
Brother/sister	4	12.0
Other	3	9.0
Don’t know	17	55.0
**Household members know adolescent’s HIV status** (*n* = 31)		
Some	20	65.0
Most all	10	32.0
Don’t know	1	6.0
**Friends know adolescent’s HIV status** (*n* = 31)		
None	29	94.0
Some	2	6.0
Most all	0	0.0
**Stigma (agree/strongly agree)** (*n* = 31)		
Although I have HIV, I am a person of worth.	27	87.0
I understand why people would reject my friendship because I have HIV.	15	48.0
When people know I have HIV I feel uncomfortable around them	13	42.0
I am embarrassed about having HIV.	11	35.0
I feel guilty about having HIV.	8	26.0
I think less of myself because I have HIV.	7	23.0
Having HIV affects whether people want to be friends with you.	6	19.0

^a^ The denominator is overall ALHIV included in the survey

### Theme 2: ART management and importance of caregivers’ support for adherence

In describing their routines around management of their ART, nearly all ALHIV described that they took their ARVs with the support of a caregiver. This caregiver support was cited as source of motivation as well as a memory aid, helping them remembering to take their ARVs:

“My grandmother and my mother [help me to take my medication]. My mother says that if I don’t take the pills, I could die or get sick.” (Filipa, 13 years).

“I take my medication alone but when I forget my mom asks me if I already took the pills.” (Miguel, 13 years).

Three participants reported taking their medications with little or no family support:

“At home no one orders me, I go [take the pills] alone when I see that the soap opera has already started at 6pm. I tell my aunt that it is time for me to take my pills, and my aunt says take it with a bit of bread or a mango.” (Isabel, 13 years).

Despite being instructed to keep their ART a secret, only two of the ALHIV reported this as being a barrier to their adherence. Forgetfulness and pill burden were the most frequently cited barriers. Additional barriers mentioned by only a few participants were difficulty taking ARVs covertly when not at home, engaging in concomitant activities and inflexibility around the time of pill intake:

“Forgetfulness and laziness … [But when this happens] I usually want to go and get the pills to take them but as the hour has passed, my sister tells me not to take it because it doesn’t help.” (Noemia, 13 years).

“Sometimes I get very tired, and I forget to take the pills …” (Sara, 12 years).

“When I’m out of the house, at someone else’s house is difficult because there are other people, and you must take the pills without being seen.” (Rui, 14 years).

When asked if they had ever stopped taking ART, two participants described their experiences.

One of the male participants, Ze, explained that he knows he acquired HIV perinatally. He lives with his aunt and grandmother because his mother stopped taking her HIV medication and died. He remembers taking his medication since he was 5 years even though he was only informed about his HIV-positive status at age 9 years. Ze always takes his medication by himself, and he also goes alone to the clinic appointments. One day he decided to stop taking his ART and it was his caregivers’ advice that convinced him to restart ART:

“Nothing [made me stop it], only spontaneous will. For one month. I started taking them [ ARVs] again because my uncle and my aunt gave me advice like if I continue to take them is for me to live, for me to continue to grow, it is to achieve my dreams.” (Ze, 13 years).

Filipa, one of our female participants, found out from a healthcare provider at the clinic that she had HIV infection when she was 13 years old. She does not know if either of her parents are living with HIV and how she acquired HIV infection. She remembers taking her medication since she was 6 years old. When she was 11 years, she got tired of taking pills without knowing why and decided to stop. Eventually, she became severely ill, and her mother discovered she had discontinued her ART:

“I didn’t know why I was taking the pills [ARVs], so one day I took the pills and went to bury them, and my mother found the pills all over the floor, and she was angry with me. [I bury them] because I didn’t like them anymore, I was tired of taking them. [I restarted taking the pills] because I got really sick. I had fevers, but it looked like malaria. My mom took me to the hospital, they did all the tests, I didn’t even move my arms, I just slept, they saw I had quitted the treatment. Then I started coming to the hospital alone.” (Filipa, 13 years).

Responding to questions related to future expectations, the majority believed that as they grow older, management of their HIV infection care and adherence to ART would become easier. These young adolescents expected that they would have greater autonomy and consequentially, they would be able to remind themselves to take their medication, allowed to carry the medication with them, and perhaps be less tired or lazy.

F: “It will be easier because my mom will leave the pills with me.

Interviewer: Why doesn’t your mother leave the pills with you now?

F: Because my sisters enter my room all the time” [and can see my medication] (Filipa, 13 years).

“It will be easier because I will control the timing of taking the pills myself”. (Miguel, 13 years).

Only survey participants who were aware of their HIV status were asked about ART management, adherence, and health services utilization (Table 3). Among these, 52% took ARVs with the support of a caregiver but the majority, 81%, went alone to the clinic for their medical appointments and ART pick-ups. Most of the participants were currently taking one ARV pill per day (71%) and one time per day (84%). A total of 45% had to miss some school time to attend clinic visits and 11% had spent at least one night in the hospital during the last year. Slightly more than a third of the participants, 35%, reported having missed at least one ARV dose in the last 30 days, with 26% indicating forgetfulness as the biggest challenge.

**Table 3:** ART management and adherence among ALHIV (*n* = 31)

	n	%
**Reports being currently on ART**	30	97.0
**Remembers age of ART initiation**	23	70.0
**Age recalled for ART initiation**, median [IQR] (*n* = 23)	10 [5–11]	
**Person responsible for administering ART** (*n* = 30)		
Self	14	45.0
Caregiver	8	26.0
Both self and caregiver	8	26.0
**Number of ARV pills taken per day**		
One ARV pill per day	22	71.0
Two or more ARV pills per day	9	29.0
**Number of times a day to take the ART**		
One	26	84.0
Two	5	16.0
**Know the names of the ARVs prescribed**	2	6.0
**Missed ART on at least one day in past 30 days**	11	35.0
**Took ART as instructed over past 30 days**		
Always	16	51.0
Almost always	12	39.0
Sometimes/usually	3	10.0
**Difficulty taking ART as instructed**		
Not hard/not very hard	29	93.0
Somewhat hard	2	6.0
**Reported reasons for any missed ART doses past 30 days**		
None missed	21	68.0
Forgot	8	26.0
Different routine	1	3.0
Unwell or vomiting	1	3.0
Fed up or tired or taking ARVs	1	3.0
**Goes to clinic appointments**		
On my own	25	81.0
With my father	6	19.0
**Have to take time off of school to attend clinic visit**	14	45.0
**Have spent at least one night in the hospital in the last year because of being too sick to go home**	7	11.0
**Viral load within 6 months of survey**	37	62.0^a^
**Viral load results** (*n* = 37)		
VL < 50 copies/mL (*n* = 37)	9	24.0
VL < 1000 copies/mL (*n* = 37)	17	46.0

^a^ The denominator is all ALHIV included in the survey

### Theme 3: high value of ALHIV peer support to overcome isolation, increase HIV literacy and to support adherence

We explored the perceived usefulness and acceptability of various types of support, including home visits, support groups and individual peer-to-peer interactions with other ALHIV. Most participants reported that they would welcome the opportunity to interact with other ALHIV to decrease their feelings of isolation and loneliness:

[They] “would help [us] not to be sad because what the others say. I could feel more secure because with them I wouldn’t feel so bad about what others say. Because of this disease, it was not me who chose it. God had some reason for me to have this disease.” (Noemia, 13 years).

Additionally, participants explained that interactions with other ALHIV would help them get answers to the many unanswered questions they have about their disease which they do not feel at ease to discuss with others. Furthermore, they would use this peer support to improve their ART adherence by having peers serve as reminders and motivators, exactly as their caregivers do:

[The peers] “could tell me how to take my medication, if I’m taking it wrongly, also let me know if I’m late taking the medication.” (Joao, 13 years).

[They would help me because] “they would tell me stories about this disease.” (Isabel, 13 years)

Nevertheless, one male participant who had received home visits and participated in support groups believed that this kind of support would be redundant because he was bored listening to the same messages about ART adherence:

:I find it a little more boring because they repeat the same words that they said before. They repeat those stories of someone who tend to fail taking his medication. They always tell me to take the pills.” (Ze, 13 years).

Responses to the HIV knowledge questions are noted in Table 4. Among 31 ALHIV who completed the HIV knowledge questions, the median percent of correct answers was 61. Even though 94% of participants were able to understand that ART lowers the amount of HIV virus in the body, only 23% understood the meaning of viral load and about a third believed you can get HIV infected by kissing someone who has HIV infection.

**Table 4:** HIV knowledge and social support among ALHIV (*n* = 61)

	n	%
**Social support (Yes)** (*n* = 61)		
Have someone who helps you when you have a problem?	55	90.0
Have someone to play with or spend time with when you feel lonely?	53	87.0
Have someone to talk to when you have questions about your HIV?	15	48.0
Have someone who makes you feel better when you are sad?	47	77.0
**HIV knowledge (Correct response)** (*n* = 31)		
It’s possible to look at a person and tell if they are HIV-positive.	22	71.0
Anyone can become infected with HIV.	22	71.0
You can get HIV by kissing a person on the mouth who is infected with HIV.	21	68.0
The amount of HIV in a person’s blood is called viral load.	12	39.0
There is a cure for HIV.	17	55.0
Antiretroviral medications (ARVs) lower the amount of HIV virus in the body.	29	94.0
It is OK to miss doses of ARVs.	26	84.0
Viral load means the amount of virus in your blood.	7	23.0
HIV cannot be passed from an HIV-positive pregnant woman to her unborn child.	10	32.0
A woman can protect her baby from getting HIV during pregnancy and breastfeeding if she takes her ARVs every day.	23	74.0
Median % of correct answers	61	

Most (90%) participants reported having someone to help them when they have a problem, but less than half (48%) reported that they could discuss HIV-related questions with others.

### Theme 4: unmet needs for sexual and reproductive health (SRH) education

We explored experiences related to romantic and sexual relationships. Half of the ALHIV interviewed had heard about other young adolescents who were dating and three knew other married or pregnant adolescents. When asked about their personal experiences, four participants (3 males, 1 female) reported that they were dating, with the three males who were in relationships also reporting that they had engaged in sexual activity:

“Interviewer: Have you ever had sex?

R: Yes, last year, with my girlfriend.

Interviewer: Besides that, have you had sex with other people?

R: Yes. With five girls. It was only last year, because when I turned 14, I stopped doing it because I heard many experiences from my friends, some started smoking, others started drinking, started leaving the house late to return the next morning, because with girls you go out at night and come back late.” (Rui, 14 years).

Participants were also asked to describe whether and how they normally accessed information about contraception, pregnancy, condom use and romantic and sexual relationships. Only two female participants indicated they had talked about these topics with a family member, and only two males explained that although they had never spoken about these issues directly, they had heard conversations from elders at home and at school. One of the female participants described how she trusted her sister to talk about SRH topics:

“She [my sister] tells me her secrets and I tell mine. In her room it is full of [contraceptive] pills, and she told me that she had a boyfriend who would take her to the hotel, and she would take the pills.” (Filipa, 13 years).

And at the same time, she described the one time her mother took the initiative to talk with her about SRH:

“My mother only said that I had to start dating only at 18, and as soon as I had a boyfriend, I should introduce him to my parents right away.” (Filipa, 13 years).

Reasons for the dearth of conversations about SRH topics with others were not clearly explained but a few participants said they assumed talking about these sensitive topics was not good for them, that they were too young, and that their parents would not approve if they confronted them with these kinds of questions. At the same time, some participants stated openly that they wished to gain more knowledge about SRH. Also, when asked how they or other adolescents would prefer to learn about these topics, they mentioned parents, siblings, friends, and healthcare providers as possible trusted sources:

Interviewer: “Would you like to know something about dating, sex, condoms, pregnancy?

J: Yes.

Interviewer: And where would you like to know more about these subjects? In your family, with doctors, on the internet, in your friends, where would you like to have information on this?

J: With my friends, because if I talked to my parents, they would tell me that I am not in the stage of knowing these things.” (Joao, 13 years).

“I would you like to know more about pregnancy, how does the person get pregnant and how to have a baby.” (Ana, 13 years).

Among survey participants, 23% reported ever have been in a romantic relationship, 10% were currently in a relationship, and 3%, both male participants, reported ever having had sex and not using condoms during the last sexual encounter (Table 5).

**Table 5:** Sexual behaviors and relationships among all ALHIV (*n* = 61)

	n	%
**Ever had a boyfriend/girlfriend**	14	23.0
**Currently in a relationship**	6	10.0
**Age of relationship partner, median [IQR]**	12 [12, 13]	
**Ever had sex**	2	3.0
**Condom used during last sex (***n* = 2)	0	0.0

## Discussion

Evidence around interventions focused on ALHIV is sparse in SSA, and even more so in Mozambique. To our knowledge, this is the first published study to focus specifically on the experiences of ALHIV in Mozambique. We explored the lived experiences of young ALHIV in northern Mozambique, from HIV status disclosure to maintenance of ART adherence, with the goal of generating evidence for interventions to improve health outcomes during this sensitive developmental phase. The experiences of the young people we talked with were along four main themes -- the beginning of a life of secrecy initiated by the HIV disclosure moment; ART management and the central role that caregivers play in adherence support; the high value that ALHIV peer support could have to overcome isolation, increase HIV literacy, and support adherence; and living with unmet needs for sexual and reproductive health education.

HIV status as a secret to be well-kept was a central theme for most participants, something imprinted into their consciousness from the moment they learned their HIV status, either through their caregivers or healthcare providers. The need for keeping HIV status a secret is understood as critical to preclude abandonment and humiliation from those close to them -- intimate friends, and family members -- as well as the general community. Secrecy around HIV infection in adolescents is an issue that has been documented in studies with ALHIV in sub-Saharan African countries such as Zambia [18], Kenya [19, 20], Tanzania [21], South Africa [22] and Malawi [23] but also in Europe and the United States [24] .

Following their caregivers’ and providers’ guidance, most participants had not yet disclosed their HIV status to any of their friends, did not knew other adolescents living with HIV or with whom they felt that they could discuss HIV-related issues. At the same time, the HIV knowledge of our participants was poor, and few could explain how they had acquired their HIV infection. These results indicate that this young population is missing out on opportunities for more social support and needs safe environments where they can discuss HIV concerns without fear. A similar situation was found in a quality of life evaluation study of adolescents with perinatal HIV infection in Ghana [25]. Hence, ALHIV peer support programs in this context could respond to needs for emotional support, offer opportunities to experience a stigma-free environment and improve HIV and ART literacy and motivation. Other studies in South Africa and Zimbabwe have demonstrated the effectiveness of peer support and adolescent-friendly services to overcome several of the most common barriers to ART adherence in ALHIV [22, 26, 27].

We have observed that HIV infection remains a highly stigmatized disease in Mozambique after decades of access to effective ART, and that healthcare providers and caregivers are perpetuating this stigma with young adolescents. This needs to be addressed openly at a policy-making level while simultaneously implementing interventions to engage the wider community to change societal HIV negative attitudes, and to protect ALHIV from being exposed to contradictory messaging of normalization and hope while promoting stigma and secrecy around living with HIV [26].

Taking daily ARVs was not described as being particularly challenging for adolescents at this young age. The biggest challenge was reported to be forgetfulness and growing tired of taking pills, commonly cited in other studies [26, 28, 29]. Medication reminders and caregiver support seem to be the main source of support for ART adherence. These findings are also consistent with studies in South Africa [29]. However, as also found in a study in Malawi [23], caregiver conversations about ART were often brief and directive and they rarely engaged the ALHIV in conversations about HIV infection transmission, but rather reinforced that HIV status was something best kept in secret.

Nevertheless, the central role that caregivers play in adherence support in young adolescents suggest that targeting programs to this young population can provide an important opportunity for early intervention. Evidence from previous studies indicate that older adolescence is a risk factor for suboptimal adherence due to various factors [30–34], but our participants expect that pill intake will become easier as they grow older and become more independent from their caregivers. Hence, programs to improve ART outcomes in this population would benefit from working on two fronts: engaging caregivers to empower them to extend their support into later adolescence [35, 36] and preparing young adolescents to develop disease management skills when caregiver support will no longer be available [37]. Long-acting ART formulations may play a particularly important role for this population when they become available.

Our study indicates that although young ALHIV in Nampula are already engaging in romantic and early sexual activities, they do not feel entitled to demand and access SRH services. Early marriage and pregnancy is a prevailing issue in the country [38, 39]; however, there is some evidence that talking openly about sex and sexuality is not common or accepted within the family setting [40]. A study with ALHIV in Tanzania also found youth perceived that they were not allowed to talk about SRH with their parents because they were too young and that caregiver communications were often in the form of warnings, not as informative discussions [41].

### Study strengths and limitations

We focused on an understudied population of young adolescents and used a mixed- methods study design.. Notwithstanding, the study had some limitations. First, we recruited purposive sample of ALHIV engaged in healthcare in three clinics and therefore cannot ascertain to what extent these findings would be valid for participants who are not engaged in care. Second, our qualitative interview sample was limited to ALHIV who were fully aware of their HIV status, but essential given questions about HIV transmission mode, disclosure and treatment. Third, although all participants were enrolled in school, we did not explore whether schooling was delayed and to what extend this delay was due to time required to manage their ART pick-ups and medical appointments. Finally, our study was conducted in an urban and semi-urban setting in one city in northern Mozambique, limiting generalization of results to ALHIV in rural contexts and other parts of the country.

## Conclusion

The findings of this study revealed the intense needs of young ALHIV in northern Mozambique. HIV-related stigma and associated secrecy continue to impose a heavy burden on and isolate ALHIV beginning in early adolescence. HIV programs should address HIV stigma openly and at all levels of intervention, including throughout the disclosure process. Of note, caregivers are central in supporting young ALHIV’s adherence to ART and their engagement should be fostered and extended to advance health outcomes of their youth. Finally, adolescent-friendly services, including HIV peer support and SRH services, will be welcomed by ALHIV and deserve prioritization in ART programs in Mozambique to prepare them for a healthy and hopeful future.

## Data Availability

Per study sponsor requirements, once the study is complete, all de-identified, archived data will be transmitted to and stored at NICHD’s Data and Specimen Hub (DASH) for use by other researchers including those outside of the study: https://dash.nichd.nih.gov/. Permission to transmit data to DASH will be included in the informed consent. Joana Falcão, the corresponding author should be contacted for requests to use study data.
